# Mouse oocytes fertilised by ICSI during *in vitro *maturation retain the ability to be activated after refertilisation in metaphase II and can generate Ca^2+ ^oscillations

**DOI:** 10.1186/1471-213X-7-72

**Published:** 2007-06-20

**Authors:** Agnieszka Jędrusik, Anna Ajduk, Paweł Pomorski, Marek Maleszewski

**Affiliations:** 1 Department of Embryology, Institute of Zoology, University of Warsaw, Warsaw, Poland; 2 Laboratory of Signal Transduction, The Nencki Institute of Experimental Biology, Warsaw, Poland

## Abstract

**Background:**

At fertilisation, mammalian oocytes are activated by oscillations of intracellular Ca^2+ ^([Ca^2+^]_i_). Phospholipase Cζ, which is introduced by fertilising spermatozoon, triggers [Ca^2+^]_i _oscillations through the generation of inositol 1,4,5-triphosphate (IP_3_), which causes Ca^2+ ^release by binding to IP_3 _receptors located on the endoplasmic reticulum (ER) of the oocyte. Ability to respond to this activating stimulus develops during meiotic maturation of the oocyte. Here we examine how the development of this ability is perturbed when a single spermatozoon is introduced into the oocyte prematurely, i.e. during oocyte maturation.

**Results:**

Mouse oocytes during maturation *in vitro *were fertilised by ICSI (intracytoplasmic sperm injection) 1 – 4 h after germinal vesicle break-down (GVBD) and were subsequently cultured until they reached metaphase II (MII) stage. At MII stage they were fertilised *in vitro *for the second time (refertilisation). We observed that refertilised oocytes underwent activation with similar frequency as control oocytes, which also went through maturation *in vitro*, but were fertilised only once at MII stage (87% and 93%, respectively). Refertilised MII oocytes were able to develop [Ca^2+^]_i _oscillations in response to penetration by spermatozoa. We found however, that they generated a lower number of transients than control oocytes. We also showed that the oocytes, which were fertilised during maturation had a similar level of MPF activity as control oocytes, which were not subjected to ICSI during maturation, but had reduced level of IP_3 _receptors.

**Conclusion:**

Mouse oocytes, which were experimentally fertilised during maturation retain the ability to generate repetitive [Ca^2+^]_i _transients, and to be activated after completion of maturation.

## Background

In all animal species studied to date the activation of the oocyte is triggered by transient elevation of the cytoplasmic concentration of free Ca^2+ ^ions ([Ca^2+^]_i_). In mammals, the increase in [Ca^2+^]_i _during fertilisation occurs in the form of [Ca^2+^]_i _oscillations, which start when the oocyte is penetrated by spermatozoon [1-5] and last for several hours until the formation of pronuclei [6]. It has been proposed that oocyte activation is caused by a soluble factor introduced into the oocyte by spermatozoon during fusion of the gametes [7-10]. More recently, a novel, spermatozoon-specific isoform of phospholipase C, named phospholipase Cζ (PLCζ), has been identified in mouse, man and cynomologus monkey, and it has been shown to be a spermatozoon-derived oocyte-activating factor [11-13].

The ability of an oocyte to be activated by fertilising spermatozoon develops during the meiotic maturation. It is known that spermatozoon-induced [Ca^2+^]_i _transients are mediated by the release of Ca^2+ ^ions from the endoplasmic reticulum through the pathway which involves inositol 1,4,5-triphosphate (IP_3_) receptors [14-17]. Oocytes develop sensitivity to IP_3 _during oocyte maturation [18-20]. The change in the organization of the endoplasmic reticulum (ER) during oocyte maturation may contribute to this enhanced sensitivity. It was demonstrated that in maturing mouse oocyte, ER undergoes reorganisation and in fully mature oocyte it aggregates within the cortical region [21]. These changes coincide with the redistribution and increase in the number of IP_3 _receptors [22].

Experimental fertilisation of immature oocytes can be used to study the development of the ability of the oocyte to be activated. Clarke and Masui [23] showed that maturing murine oocytes, which had been penetrated by spermatozoa, were able to complete meiotic maturation, but did not undergo subsequent activation. This demonstrates that spermatozoon-derived oocyte-activating factor is unable to induce activation when introduced into maturing oocyte, probably because of its inactivation by the cytoplasm of maturing oocyte [24]. Immature oocyte are able to create [Ca^2+^]_i _transients soon after being penetrated by spermatozoon [25], but they generate fewer [Ca^2+^]_i _oscillations and cease oscillating earlier than mature oocytes [25]. Tang et al. [26] demonstrated that *in vitro *matured oocytes lost the ability to generate [Ca^2+^]_i _transients induced by sperm-factor when they had been stimulated by sperm extracts during oocyte maturation. These authors suggested that in mouse oocytes, the prolonged [Ca^2+^]_i _oscillations depend on the "maternal machinery" mechanism that can be switched on only once and becomes inactivated by premature introduction of the spermatozoon-derived oocyte-activating factor [26]. However, another possibility is that the changes in Ca^2+ ^signaling pathway which take place after its premature stimulation, are quantitative in nature, and depend on the amount of the sperm factor introduced into the oocyte. Thus, the aim of our experiments was to examine if the premature introduction of a single spermatozoon into maturing oocyte, affects its ability to be activated and to generate Ca^2+ ^response after completion of maturation, in metaphase II (MII) stage. Since mouse oocytes fertilised during maturation *in vitro *lose their ability to fuse with additional spermatozoa when they reach MII stage [27], we used intracytoplasmic sperm injection (ICSI) to introduce spermatozoa into maturing oocytes. Next, when the oocytes reached MII we refertilised them *in vitro*. We observed that oocytes injected with the spermatozoa during *in vitro *maturation, retained, after completion of maturation, the ability to be activated and to develop [Ca^2+^]_i _oscillations in response to penetration by spermatozoa.

## Results

### Efficiency of ICSI into maturing oocytes

Injection of spermatozoon does not affect the ability of the oocytes to complete maturation; 83% of injected and 83% of control (uninjected) oocytes extruded PB1 and reached MII stage. In order to assess the efficiency of ICSI into maturing oocytes, the oocytes were fixed between 0.5 and 5 h after injection of spermatozoa and after completion of meiotic maturation (12 – 18 h after ICSI) and observed as a whole-mount preparations. The chromatin of spermatozoa was present in the cytoplasm of 88% (37/42) of oocytes, which were fixed 0.5 – 5 h after ICSI. Nuclei of spermatozoa, which were injected into oocytes, first underwent decondensation and then recondensed. These changes were similar to spermatozoon transformations observed during fertilisation of mature MII oocytes [28]. However, when spermatozoa-injected oocytes were examined after completion of maturation, only 65% (21/32) of oocytes contained in their cytoplasm the recondensed spermatozoon-derived chromatin. The proportion of oocytes, which had spermatozoa in their cytoplasm at the end of maturation (12 – 18 h after ICSI), was significantly lower (p < 0.05) than the proportion of oocytes in which the presence of the spermatozoon-derived chromatin was observed 0.5 – 5 h after ICSI. This suggested that some maturing oocytes discarded the microinjected spermatozoa. This was confirmed by our finding that half (6/11) of the oocytes subjected to ICSI and lacking the spermatozoon-derived chromatin in their cytoplasm had, in addition to first polar body (PB1), the "pseudo-polar bodies" containing recondensed chromatin of the spermatozoon, and another half of the oocytes (5/11) had noticeably larger first polar body that, besides the meiotic chromosomes, also contained the spermatozoon-derived chromatin.

### Can oocytes fertilised during maturation be activated after refertilization in MII?

**Figure 1:**
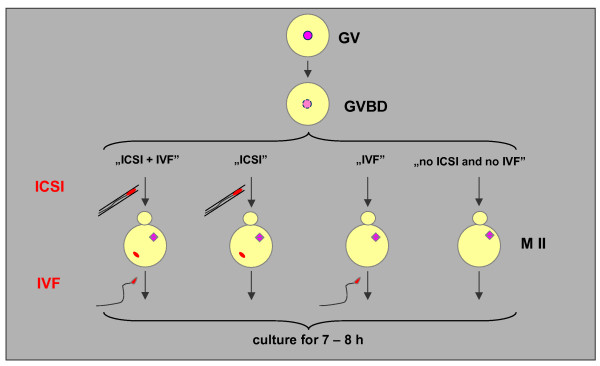
Design of the experiment (see description in text).

Experimental oocytes (variant "ICSI + IVF") were fertilised twice: first by ICSI 1 – 4 h after GVBD and next by conventional IVF in MII stage. There were three control groups of maturing *in vitro *oocytes: 1) fertilised once by ICSI during maturation, 1 – 4 h after GVBD (variant "ICSI"), 2) fertilized once by conventional IVF at MII stage (variant "IVF"), and 3) unfertlised oocytes which matured *in vitro *up to MII stage ("no ICSI and no IVF" variant). Oocytes from all three variants were cultured for 7 – 8 h and were examined for the signs of activation (i.e. extrusion of the second polar body (PB2) and formation of pronuclei). Subsequently, the oocytes were fixed and mounted on slides. The diagram of the experiment is shown in Figure 1.

**Figure 2:**
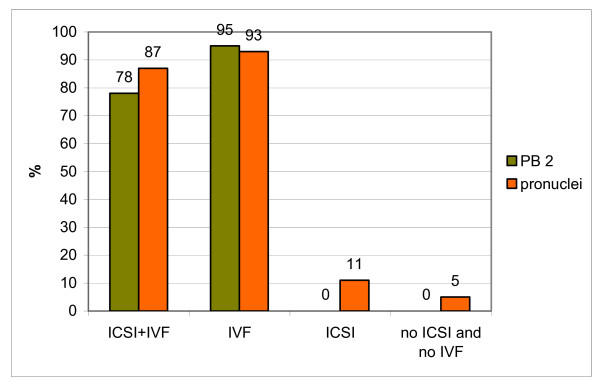
Frequency of the activation of oocytes in different experimental variants. See text for the description of the variants of the experiment. Differences between "ICSI + IVF" and "ICSI" variants, as well as between "IVF" and "no ICSI and no IVF" variants are statistically significant (p < 0.05).

The number of activated oocytes in various variants of the experiment is presented in Table 1 and in Figure 2. In a double fertilisation variant ("ICSI + IVF") the extrusion of the PB2 was observed in 78% of oocytes and 87% of oocytes had 1–4 pronuclei. The presence of one or two pronuclei indicated that either the first (introduced by ICSI) spermatozoon-derived nucleus or the second, (introduced by IVF) or both (introduced by ICSI and IVF) spermatozoa-derived nuclei have been eliminated from the oocyte (see above). Another possibility was that the chromatin of microinjected spermatozoon had merged with chromosomes of the oocyte during maturation and, thus, after activation, formed a single hybrid pronucleus [29]. The possibility that the formation of a single pronucleus resulted from spontaneous activation of the oocyte was tested in control experiments described below. The presence of more than three pronuclei in activated oocytes indicated polispermic penetration during IVF.

**Table 1:** Frequency of activation of *in vitro *maturing oocytes which were fertilised, during maturation, by ISCI, 1 – 4 h after GVBD, refertilized at metaphase II (ICSI + IVF) and were fixed for cytological examination 7 – 8 h after refertilisation. Control, *in vitro *maturing oocytes were inseminated once: only by ICSI during maturation (ICSI), only in MII stage (IVF) or were not inseminated at all (no ICSI and no IVF).

	**Proportion (%) of oocytes with**	
	
	**2 PB**	**pronuclei**
**ICSI + IVF**	29/39 (78)^a^	27/31 (87)^c^
**IVF**	35/37 (95)^a^	25/27 (93)^c^
**ICSI**	0/27 (0)^b^	3/27 (11)^d^
**No ICSI and no IVF**	0/45 (0)^b^	2/37 (5)^d^

^a, b ^and ^c, d ^Values with different superscripts are significantly different (p < 0.05).

Among oocytes, which matured *in vitro* and were fertilised only once at the MII stage ("IVF" variant), almost all extruded PB2 and formed pronuclei (95% and 93% respectively). Oocytes from both groups ("ICSI + IVF" and "IVF") extruded PB2s approximately 1 – 2 h after the end of incubation with spermatozoa. Fertilised oocytes from "IVF" group contained two to seven pronuclei. Presence of two pronuclei indicated monospermic penetration, while the presence of more than two pronuclei in the oocyte cytoplasm suggested polyspermic fertilization.

None of the oocytes from "ICSI" and "no ICSI and no IVF" groups extruded the PB2 during culture. 95% of oocytes from "no ICSI and no IVF" group were blocked in MII and only 2 oocytes from this group underwent spontaneous activation and formed a single pronucleus without extruding PB2s. 89% of oocytes from "ICSI" group did not undergo activation, despite the presence of the spermatozoon-derived chromatin in their cytoplasm.

### Are the oocytes fertilised during maturation able to respond with [Ca^2+^]_i _transients to spermatozoa penetration in MII?

**Figure 3:**
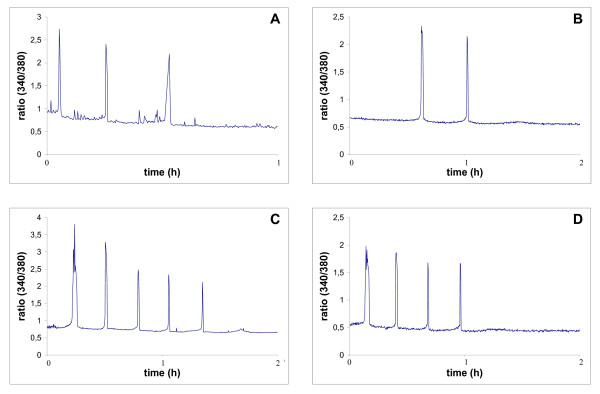
[Ca2+]_i _oscillations, measured as the ratio of Fura-2 fluorescence excited sequentially at 340 and 380 nm every 10 s. (A) maturing oocyte fertilised with ICSI. Due to technical reasons the first [Ca^2+^]_i _spike was not recorded. (B) MII oocyte, which was fertilised by ICSI during maturation and was refertilised after completion of the maturation, (C) MII oocyte, which during maturation was subjected to sham microinjection and was fertilised after completion of the maturation, (D) MII oocyte, which matured *in vitro*, and was fertilised at the end of maturation.

**Figure 4:**
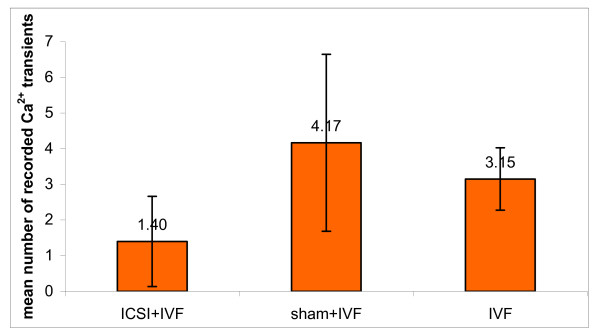
Mean number of [Ca^2+^]_i _transients generated in MII oocytes in response to spermatozoa during 60 min after fertilization. Differences between "ICSI + IVF" variant and "sham+IVF" and "IVF" variants are statistically significant (p < 0.05).

First we examined whether maturing oocytes were able to respond with [Ca^2+^]_i _oscillations to the injection of spermatozoon. We found that 80% of injected oocytes generated repetitive [Ca^2+^]_i _transients (in one oocyte only one [Ca^2+^]_i _peak was recorded; however, because of technical reasons we were unable to capture the first [Ca^2+^]_i _spike in any of examined oocytes, we assume that this oocyte produced at least 2 [Ca^2+^]_i _spikes) (Figure 3A). Next we tested whether oocytes fertilised during maturation can respond with [Ca^2+^]_i _rise after insemination at MII. We found that majority of the oocytes from "ICSI + IVF" experimental variant generated Ca^2+ ^response after refertilisation (7/10) and half of them (5/10) generated 2–3 [Ca^2+^]_i _spikes during first hour after sperm penetration (the first [Ca^2+^]_i _spike was considered as occurring at the time of sperm entry) (Figure 3B, Table 2). The mean number of [Ca^2+^]_i _transients was 1.4 +/- 1.3 (Figure 4). In "IVF" and "sham+IVF" control groups most of the oocytes produced at least 3 [Ca^2+^]_i _transients (Table 2 and Figure 3C,D). The mean number of [Ca^2+^]_i _spikes in both control groups ("IVF": 3.2 +/- 0.9 and "sham + IVF": 4.2 +/- 2.5) was significantly higher than in "ICSI+IVF" group (p < 0.05) (Figure 4).

**Table 2:** Number of [Ca^2+^]_i _transients generated in M II oocytes in response to spermatozoa during 60 min from fertilization.

	**Proportion (%) of oocytes in which given number of [Ca^2+^]_i _transients were observed:**					
	
	**0**	**1**	**2**	**3**	**4**	**>4**
**ICSI + IVF**	3/10 (30)	2/10 (20)	4/10 (40)	1/10 (10)	0	0
**IVF**	0	0	5/20 (25)	8/20 (40)	6/20 (30)	1/20 (5)
**sham + IVF**	0	0	2/12 (17)	5/12 (42)	1/12 (8)	4/12 (33)

### Is the IP_3 _receptor downregulated in oocytes fertilised during maturation?

Lower number of [Ca^2+^]_i _transients observed in oocytes, which were fertilised during maturation and refertilised at MII could result from the IP_3 _receptor downregulation caused by introduction of spermatozoon into maturing oocyte. To test this possibility we compared the level of IP_3 _receptor between MII oocytes, which were or were not fertilised during maturation *in vitro*, by Western blot technique. Due to relatively low efficiency of ICSI into maturing oocytes, in this experiment we used conventional IVF to introduce spermatozoa into oocytes.

**Figure 5:**
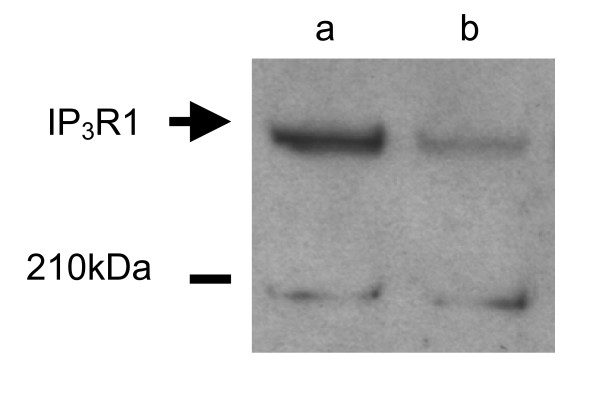
Western blot analysis of IP_3 _receptor in mouse oocytes which matured *in vitro*, and were fertilised during maturation, and in *in vitro *matured oocytes, which were not fertilised. (a) in vitro matured oocytes in MII stage, 100 oocytes per lane, (b) oocytes fertilized during in vitro maturation, which achieved MII stage, 100 oocytes per lane.

We observed that in MII oocytes penetrated by a single spermatozoon during maturation *in vitro*, the level of IP_3 _receptor was lower than in uninseminated control oocytes (Figure 5).

### Do the oocytes fertilised during maturation have a lower level of MPF activity?

We found that oocytes fertilised during maturation and refertilised at MII underwent activation in spite of lower number of [Ca^2+^]_i _transients accompanying refertilisation. There was a possibility that introduction of spermatozoon into maturing oocyte resulted in partial decline of the MPF activity that allowed oocyte to respond to a weaker activating stimulus. Since MPF activity can be quantified using a histone H1 kinase assay [30,31], we compared the level of histone H1 kinase activity between MII oocytes which were or were not fertilised during maturation *in vitro*. In this experiment we also used *in vitro* fertilisation instead of ICSI to introduce spermatozoa into maturing oocyte

**Figure 6:**
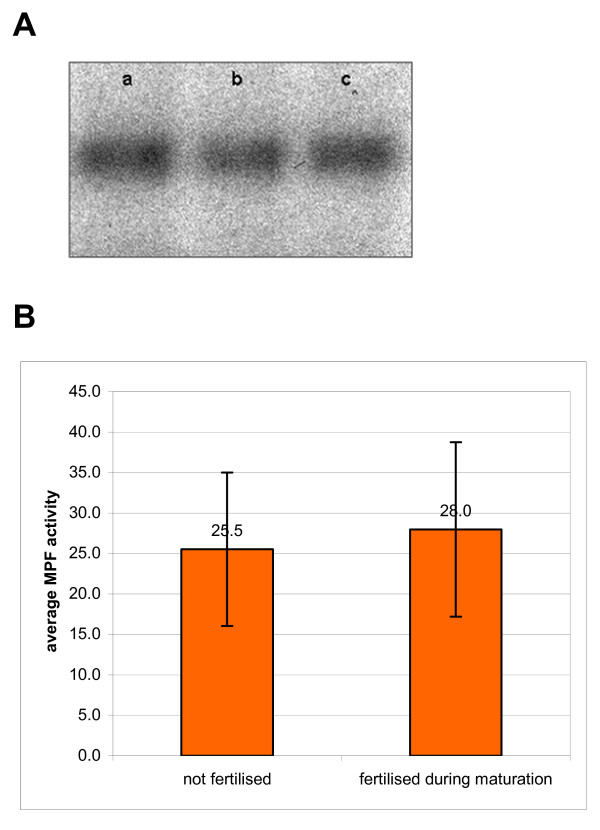
Histone H1 kinase activity in MII oocytes, which matured *in vitro*, and were fertilised during maturation, and in *in vitro *matured oocytes, which were not fertilised. (A) autoradiography showing the intensity of incorporation of [^32^P] into exogenous H1 histone in lysates prepared from MII oocytes. Each line corresponds to the activity prepared from 5 oocytes. (a) control, uninseminated oocytes, (b, c) oocytes, which were fertilised during maturation, 1 – 4 h after GVBD. (B) mean activity of histone H1 kinase in MII oocytes, which were fertilised during maturation and in MII oocytes, which were not fertilised. Differences between these two groups are not statistically significant (p > 0.05).

We found that the activity of histone H1 kinase in MII oocytes penetrated by a single spermatozoon during maturation *in vitro *and in uninseminated control oocytes was approximately the same (respectively 28.0 ± 10.8 and 25.5 ± 9.5 arbitrary units, expressed as a relative average intensity of histone H1 phosphorylation, Figure 6).

## Discussion

### Oocytes fertilised during maturation can be activated after refertilisation in MII and can respond to spermatozoa with [Ca^2+^]_i _transients

The ability of the oocyte to be activated by spermatozoa requires a calcium signalling system, which develops during oocyte maturation. In ovulated mature oocytes spermatozoa trigger [Ca^2+^]_i _oscillations that result in oocyte activation [1-5]. One of the methods used to study how the ability of the oocyte to respond to activating spermatozoa develops during maturation, is the examination of the oocyte response to experimental, premature fertilization. It was demonstrated previously that immature oocytes are capable of producing [Ca^2+^]_i _transients after penetration by spermatozoon, but they generate fewer [Ca^2+^]_i _oscillations and cease oscillating earlier than mature oocytes [25]. In our present study we investigated whether oocytes that were fertilised by ICSI during maturation, retain the ability to be activated when refertilised after the completion of the maturation. We also examined if they can respond to refertilisation with [Ca^2+^]_i _oscillations. We have shown that MII oocytes, which were subjected to double fertilisation, are able to generate spermatozoa-induced [Ca^2+^]_i _increase and to undergo activation. The frequency of their activation (i.e. completion of meiotic division and formation of pronuclei) was similar to the frequency of activation of oocytes that were fertilised only once, at MII stage. Moreover, 50% of the oocytes, which were refertilised, reacted to the penetration by spermatozoa by generating 2 or 3 [Ca^2+^]_i _transients. The fact that not all of refertilised oocytes which underwent activation generated [Ca^2+^]_i _oscillations, should not be regarded as contradictory, since it is known that a monotonic rise in [Ca^2+^]_i _is sufficient to activate aged oocytes [32-34]

Similar type of experiment was performed previously by Tang et al. [26]. However, these authors microinjected mouse oocytes during maturation with bovine sperm extracts, rather than with whole spermatozoa. They demonstrated that such oocytes cannot generate [Ca^2+^]_i _oscillations in response to second injection with the sperm extract performed after completion of maturation. These oocytes produced only a single [Ca^2+^]_i _transient. We believe that the Ca^2+ ^response of refertilised oocytes observed in our experiments was closer to response of the oocyte during natural fertilisation than that observed by Tang et al. [26] in their experiments. This difference can be partially explained by the fact, that Tang et al. [26] injected oocytes with an amount of sperm extract which was an equivalent of approximately 2 – 3 spermatozoa/oocyte and we injected each oocyte with a single spermatozoon, which introduced a smaller amount of sperm-derived, oocyte-activating factor. Studies examining the effect of polyspermy [35] and the effect of different quantity of microinjected PLCζ mRNA [12] on oocyte Ca^2+ ^response showed positive correlation between the quantity of sperm activating factor introduced and the frequency of [Ca^2+^]_i _oscillations. It is possible that the introduction of larger than normal i.e., as it happens during monospermic fertlisation, quantity of sperm activating factor causes stronger than normal inactivation of IP_3 _receptors [14,36] and in consequence lowers the oocyte Ca^2+ ^releasing ability and eliminates [Ca^2+^]_i _oscillations. Another reason for the discrepancy between our results and the observations made by Tang et al. [26] could be related to differences in the amounts of activating factor in spermatozoa of different species. It was demonstrated previously that protein extracts prepared from spermatozoa of different species induce [Ca^2+^]_i _oscillations of untypical parameters in human [37] and mouse [38] oocytes. Tang et al. [26] used sperm extracts prepared from bovine spermatozoa to induce Ca^2+ ^response in mouse oocytes. There is a possibility that bovine sperm extract induces stronger stimulation, and in consequence, stronger inactivation of IP_3 _receptors than activating factor from a single mouse spermatozoon, and results in a weaker Ca^2+ ^response after repeated microinjection with sperm extract. Thus the conclusion of Tang et al. [26] that [Ca^2+^]_i _oscillations are dependent on the mechanism that functions only once in mouse oocytes and is abolished by sperm-derived oocyte-activating factor is probably applicable only to their experimental system and is not universally correct.

### Refertilisation of oocytes fertilised during maturation generates lower number [Ca^2+^]_i _transients than in oocytes fertilised only once

We observed that at least some oocytes, which were refertilised, were able to generate more than one [Ca^2+^]_i _transient as a reaction to penetration by spermatozoa. However, number of transients in these oocytes was significantly lower than in oocytes that were fertilised only once at MII. The most likely explanation of this observation is that oocytes, which were fertilised during maturation had a lower number of IP_3 _receptors present in ER that normal mature oocytes. As it was already mentioned above, it was shown that IP_3 _receptors are downregulated after fertilisation by proteolytic degradation [14,36]. During fertilisation an intense production of IP_3 _is observed due to the activity of spermatozoon derived phospholipase Cζ [13,39-41]. Binding of IP_3 _to IP_3 _receptors causes Ca^2+ ^release and subsequently leads to the degradation of IP_3 _receptors by the proteasome [14]. The loss of IP_3 _receptors was also observed in immature oocytes upon penetration by spermatozoa, which demonstrates that degradation of IP_3 _receptors does not require the normal events associated with oocyte activation [36]. We also performed a preliminary experiment on the effect of the sperm penetration during maturation on the level of IP_3 _receptors in oocytes. We found that at MII stage oocytes fertilised during maturation had much less IP_3 _receptors than uninseminated control oocytes. The role of the degradation of IP_3 _receptors in regulation of [Ca^2+^]_i _transients during fertilisation was demonstrated by Brind et al. [36] who exposed maturing oocytes to a potent IP_3 _receptor agonist adenophostin A. In these oocytes adenophostin caused downregulation of IP_3 _receptors to a greater extent than spermatozoa because its 100-fold greater potency than IP_3 _and its resistance to IP_3_-metabolising enzymes [42]. The oocyte response to the adenophostin was dose dependent: oocytes treated with low dose responded to fertilisation at MII stage with only one [Ca^2+^]_i _transient and oocytes exposed during maturation to higher concentration of adenophostin had their Ca^2+ ^signalling completely abolished [36]. These results strongly support our conclusion that the downregulation of IP_3 _receptors in immature oocytes after ICSI was responsible for the reduced [Ca^2+^]_i _oscillations during refertilisation.

### Activation of refertilised oocytes, which were fertilised during maturation, does not result from the lower level of MPF activity

The increase of [Ca^2+^]_i _that occurs during fertilisation leads to the degradation of M-phase cyclins and in consequence to the drop of the activity of MPF kinase and to the exit of oocytes from the MII block [43-45]. Our present results demonstrate that refertilised oocytes undergo activation despite the lower, than in normal fertilization, number of [Ca^2+^]_i _transients generated by refertilising spermatozoon. At first we suspected that fertilisation of oocytes during maturation could lower the level of MPF activity in these oocytes. However, we found that this was not the case; matured *in vitro *oocytes, which were fertilised during maturation had similar levels of MPF activity to those of unfertilized oocytes. Thus, the MPF activity of MII oocyte that developed from the oocyte fertilised during maturation, is not affected by the premature fertilisation.

Kubiak [33] has shown that after ovulation, the MII oocytes gradually develop the ability for activation. Soon after reaching MII stage (13 h after the administration of hCG) oocytes were able to be activated by the sperm, but not by the parthenogenetic factor (ethanol), which is known to generate only a single [Ca^2+^]_i _transient [46]. However, the activation of MII oocytes, which were isolated 16 – 17.5 h after hCG, was induced with equal efficiency by [Ca^2+^]_i _oscillations (induced by spermatozoa) and by a single [Ca^2+^]_i _transient (produced by ethanol) [33]. In our experiments MII oocytes, which completed maturation *in vitro*, were refertilised approximately 18 h after hCG, i.e at the time when prolonged [Ca^2+^]_i _oscillations most likely are no longer necessary for full activation.

In summary, we have demonstrated that when maturing mouse oocytes were fertilised by ICSI, they did not develop a membrane block to the polyspermy, and could be refertilised at MII. Oocytes, which were refertilised underwent activation, extruded PB2s and formed pronuclei with similar frequency as control *in vitro *matured oocytes, which were fertilised only once at MII stage. Activation after refertilisation was accompanied by Ca^2+ ^response, which in 50% of oocytes had a form of [Ca^2+^]_i _oscillations. However, the number of [Ca^2+^]_i _transients observed in refertilised MII oocytes was significantly lower than in oocytes fertilised only once at MII.

## Conclusion

We demonstrated that in mouse oocytes the Ca^2+ ^signalling pathway that is responsible for generation of [Ca^2+^]_i _oscillations during fertilisation and for activation of the oocyte, is not completely inactivated after premature penetration by spermatozoon. Thus, we have shown that in mouse oocyte, the molecular mechanism, which is involved in oscillatory Ca^2+ ^response to fertilisation can be switched on more than once.

## Methods

### Animals

F1 (C57Bl/6 × CBA/H and CBA/H × C57Bl/6) female (4 – 12 weeks old) and male (4 – 6 months old) mice were used for the experiments. Animal studies were approved by Local Ethic Committee No.1 in Warsaw, Poland, according to the European Union Council Directive 86/609/EEC of 24 November 1986 on the approximation of laws, regulations and administrative provisions of the member states regarding the protection of animals used for experimental and other scientific purposes [47,48]. All animals were raised on the premises.

### Chemicals, media and conditions of *in vitro *culture of oocytes

All chemicals, unless otherwise stated, were obtained from Sigma-Aldrich Sp. z o.o. (Poznan, Poland). Medium M2 (medium 16 buffered with HEPES [49]) containing bovine serum albumin (BSA, 4 mg/ml) was used for collection of immature oocytes, MII oocytes and for culture of MII oocytes inseminated *in vitro*. *In vitro *maturation of oocytes was carried out in DMEM. For ICSI, spermatozoa were suspended in M2 (without BSA) supplemented with pirovinylopirolidone (PVP MW 360000, 12% solution). Microinjection of spermatozoa was carried out in M2 (without BSA) supplemented with polyvinyl alcohol (PVA, 0.1 mg/ml). For *in vitro *fertilization spermatozoa were capacitated in fertilization medium [50] containing BSA (4 mg/ml). *In vitro *fertilization was performed in the same medium. Fertilization medium was preincubated before the beginning of experiment for about 24 h at 37.5°C in a humidified atmosphere of 5% CO_2 _in air. *In vitro *culture was carried out in droplets of medium under mineral oil in plastic dishes (35 × 10 mm, Falcon, Becton Dickinson, USA) at 37.5°C in a humidified atmosphere of 5% CO_2 _in air.

### Obtaining of germinal vesicle (GV, immature) oocytes

Follicular development was stimulated in F1 female mice by an intraperitoneal injection of 10 IU of pregnant mare serum gonadotropin (PMSG, Folligon, Intervet, Netherlands). Forty-seven to fifty-one hour later females were killed by cervical dislocation. Fully grown oocytes were released from ovarian antral follicles punctured with a needle into M2. Oocytes were freed from cumulus cells by pipetting, and were cultured in M2 for 2 h. Only oocytes that during that time underwent germinal vesicle breakdown (GVDB) were used for further manipulations.

### ICSI procedure

One to four hours after GVBD oocytes were injected with spermatozoa according to the method of Kimura and Yanagimachi [51]. A piezo-driven micropipette (MW Piezo Stepper PM 10-1, Leica, Germany) was used. Micromanipulations were performed under an inverted microscope (Diaphot 300, Nikon, Japan) equipped with Hoffman modulation contrast optics. The microscope stage was cooled with a temperature-controlling plate (Semic Bioelektronika, Kraków, Poland) to about 12–14°C. One hour before microinjection spermatozoa were released from *caudae epididymides *of F1 male mice into M2. After dispersal of the spermatozoa, a small volume (~1 μl) of the suspension was transferred to a droplet of M2 without BSA supplemented with PVP. Oocytes were placed in a droplet of M2 without BSA supplemented with PVA and in the same droplet the microinjection was performed. Several piezo pulses (speed 100 mm/s, step size 1 μm) were given to allow *zona pellucida *penetration. The oolemma was perforated with 2 – 3 piezo pulses and the spermatozoon was introduced into the oocyte. Injected oocytes were kept for 10 min in micromanipulation medium on the cooled stage. They were then transferred to M2 for 10 min at room temperature and subsequently incubated in DMEM at 37.5°C under 5% CO_2_. On average it took 5 – 10 min to inject spermatozoa into a group of 5 – 6 oocytes. During one experiment microinjection was carried out for 2 – 3 h.

### *In vitro *maturation of oocytes

Oocytes injected with spermatozoa and uninjected control oocytes were placed in small droplets of DMEM and cultured for 12 – 14 h. Oocytes were subsequently examined under inverted microscope (Labovert FS, Leitz, Germany). Only oocytes, which extruded the first polar body (PB1), were selected for further procedure.

### *In vitro* fertilisation

*Zonae pellucidae *were removed by exposure of oocytes to acidic Tyrode's solution (pH 2.5; [52]). Next, the oocytes were washed in M2 and transferred to M2 for 30 min incubation, subsequently oocytes were fertilised *in vitro*. Spermatozoa from *caudae epididymides *of a mature F1 male mouse were suspended in 0.5 ml of fertilization medium and incubated for 1.5 h to allow capacitation and spontaneous acrosome reaction. Concentration of spermatozoa was approximately 2 × 10^7 ^spermatozoa/ml. *Zona *free oocytes were placed in 100 μl droplets of fertilisation medium and 1 μl of the preincubated suspension of spermatozoa was added (final concentration was approximately 2 × 10^2 ^spermatozoa/ml). Control oocytes were incubated in fertilisation medium without spermatozoa. 30 min after the insemination, oocytes were transferred to M2 and were gently pipetted several times to remove loosely attached spermatozoa. Subsequently oocytes were cultured in M2 for 7 – 8 h. Cultured oocytes were examined under inverted microscope at 40 min intervals, for the signs of activation (i.e. extrusion of the second polar body (2 PB) and pronuclear formation).

### Cytological examination

Seven – eight hours after the insemination oocytes were fixed with Heidenhein's fixative. Whole-mount preparations were stained with haematoxylin according to the method of Tarkowski and Wróblewska [53].

### Measurement of intracellular Ca^2+^

To monitor changes in the level of [Ca^2+^]_i _the oocytes microinjected with spermatozoa during maturation *in vitro *were cultured until they reached MII stage. Subsequently, oocytes were loaded with the Ca^2+^-sensitive fluorescent dye, Fura-2 AM (Molecular Probes, Leiden, Netherlands). For loading, oocytes were incubated in 2 μm Fura-2 AM in M2 for 30 min as described by Kline and Kline [54,55]. After loading the oocytes were washed in M2 and *zonae pellucidae *were removed with acidic Tyrode's solution. Next oocytes were transferred to a heated (37°C) chamber (Chance proper LTD, Smethwick, Warley, UK) containing M2 without BSA and 1 μl of the suspension of capacitated spermatozoa was added (except maturing oocytes in which [Ca^2+^]_i _oscillations were examined after ICSI). Chamber was put on the stage of inverted microscope (Diaphot, Nikon, Japan). Fura-2 was excited sequentially at 340 and 380 nm every 10 s, and the fluorescence was collected using 10× objective. The emitted light was passed through a long pass filter (510 nm) and was gathered using PCC (Photon Counting Camera, Retiga 1300, Q Imaging, Burnaby, Canada). Oocytes were observed for 2 h. After this time fluorescent signal corresponding to [Ca^2+^]_i _transients ceased even in control MII oocytes, which were supposed to generate long-lasting [Ca2+]i oscillations. This was probably caused by compartmentalisation of the Fura stain. Data was analyzed with AQM 6.0 software (Kinetic Imaging LTD, Liverpool, UK).

### Histone H1 kinase assay

Activity of MPF was calcuated as an activity of kinase of histone H1 according to the method described by Verlhac et al. [56], in MII oocytes, which during maturation *in vitro *were inseminated approximately 3 h after GVBD. Preparation of oocytes and spermatozoa and *in vitro *insemination were carried out as described above. After insemination oocytes were cultured in DMEM for 12 – 14 h and only oocytes, which extruded the first polar body (PB1), were selected for MPF assay. To select monospermic oocytes from polyspermic ones, oocytes were incubated in Hoechst 33342 dye solution (100 ng/ml of M2) to stain chromatin. Next, the oocytes were washed in M2 and were transferred into drops of M2 in glass-bottom dish (WillCo-dish, WillCo Wells BV, Amsterdam, Netherlands). Oocytes were examined under inverted epifluorescence microscope (Axiovert 135, Carl Zeiss, Germany). Only oocytes in which only one group of spermatozoon derived chromatin was visible were selected, washed in PBS and pooled in groups of 5 in 1 μl drops of PBS. Samples were frozen and stored in -80°C.

3 μl of lyses buffer (containing 0.16 M glicerophosphate, 40 mM EGTA (pH 7.3), 30 mM MgCl2, 2 mM DTT, protease inhibitor (diluted 1:20, Complete Protease Inhibitor Cocktail, Roche, Germany), and BSA (11.3 mg/ml); final concentrations) was added to each sample. Oocytes were then lysed by freezing and thawing, and subsequently 1.5 μl of reaction buffer (containing 0.5 mg/ml histone H1, 5 mM ATP and 1.67 μCi/μl [^32^P]-ATP; final concentrations) was added. Samples were incubated 30 min in 30°C. The reaction was stopped by addition of Laemmli buffer [57]. Samples were boiled for 10 min and proceeded for 12% SDS-PAGE. Gels were exposed to autoradiography films at -80°C for 24–72 h. Intensity of bands on autoradiography films was measured with GelDoc using software Quantity One 4.2.2. (Bio-rad, Hercules, Canada). It reflected intensity of histone H1 phosphorylation and enabled us to quantify histone H1 kinase (MPF) activity. The experiment was repeated eight times.

### Examination of the level of IP_3 _receptors

The level of IP_3 _receptor was examined in monospermic MII oocytes, which were obtained according to the procedure described above for the histone H1 assay. Cell lysates from 100 oocytes were mixed with 4× NuPage LDS sample Buffer and 10× NuPage Sample Reducing Agent (Invitrogen, Carlsbad, CA, USA) and were heated for 10 min in 70°C. The samples were subjected to NuPage Novex 3–8% Tris-Acetate gels (Invitrogen, Carlsbad, CA, USA) and separated proteins were transferred onto PVDF membranes (Hyperbond-P, Amersham Biosciences, Little Chalfont Buckinghamshire, UK), which were probed with a rabbit polyclonal antibody (Rbt03) raised against a 15 amino acid peptide sequence of the C-terminal end of the IP3 receptor-1 subtype [58] diluted 1:500 in 5% non-fat milk in TTBS. A goat anti-rabbit antibody (Pierce, Rockford, IL, USA) conjugated with horseradish peroxidase diluted 1:7000 was used as a secondary antibody in 1 h incubation. Detection was performed by the enhanced chemiluminescence technique using SuperSignal West Dura Extended Duration Substrate reagents (Pierce) according to manufacturer's instruction. The experiment was performed three times.

### Photographic documentation and statistical analysis

Oocytes were photographed in the microscope equipped with a digital camera (Coolpix 995, Nikon, Japan). Statistical analysis of results was performed using Fisher's exact test and t-Student's test.

## Authors' contributions

AJ carried out ICSI and participated in IVF experiments, in Ca^2+ ^studies and in drafting the manuscript. AA carried out IVF experiments, the examination of MPF activity and examination of IP_3 _receptor degradation, participated in Ca^2+ ^studies and in drafting the manuscript. AJ and AA contributed equally to this work. PP carried out Ca^2+ ^studies. MM conceived of the study, participated in its design and coordination and drafted the manuscript. All authors read and approved the final manuscript.
